# Sanitary Legislation

**Published:** 1856-10

**Authors:** 


### SANITARY LEGISLATION.

### SALE OF BAD MEAT IN LONDON.

A COMMITTEE appointed by the Metropolitan Association of Medical Officers of Health have issued a report on the subject of unwholesome meat. The report shows:—1. That large quantities of unwholesome meat are sold in London. In the year 1855, 26 live animals, 612 entire carcasses, 696 quarters, 8 sides, and 227 joints of beef, mutton, veal, and lamb were seized as bad in the city, besides great numbers of poultry and game. But much meat which could not be sold in the city is sold in the suburbs. 2. That the signs of bad meat are *colour*, which is either dingy or too bright; *smell*, which is peculiarly sour and sickening; and *a decided wetness of the meat and soft flabbiness*. 3. That there are also special signs of disease. 4. That illnesses are produced by the eating of bad meat, as tapeworm from measly pork, and poisoning from unsound meat badly cooked.

The subject discussed by the Committee is most important; but we must, in all friendliness, say, that if the members of the Committee wish to direct public attention seriously to so great an evil, they must produce a much more vigorous, scientific, and concise report than the one here noticed, which, in plain truth, and notwithstanding the excellence of the names attached to it, is a loose, hastily concocted, and therefore weak production.

To those interested in the subject of diseased animal food, we would direct attention to a series of replies by M. Soumille of Avignon, to certain inquiries issued in 1854 by the Imperial and Central Society of Veterinary Medicine of France. See *Gazette des Hôpitaux*, October 14th, 1854; and for an abstract, JOURNAL OF PUBLIC HEALTH, April 1855.

### THE CENSUS OF IRELAND.

PARTS V and VI of the *Census of Ireland* are now published in three massive volumes. The first volume of the fifth part contains a history of Epidemic Pestilences in Ireland. For profound historical research, it would be hard to find anything that equals this interesting document. In this section of the Journal it would be impossible to enter into an analysis of the labours of the Census Commissioners; but we only reserve the labour for a more fitting opportunity.

### REPORT ON THE ADULTERATION OF FOOD.

The final Report of the Select Committee of the House of Commons, appointed to inquire into the adulteration of food, drinks, and drugs, contains many valuable statements and suggestions. The following are amongst the most important in a practical point of view.

"Not only is the public health exposed to danger, and pecuniary fraud committed on the whole community; but the public morality is tainted, and the high commercial character of this country seriously lowered, both at home and in the eyes of foreign countries. Though, happily, very many refuse, under every temptation, to falsify the quality of their wares, there are, unfortunately, large numbers who, though reluctantly practising deception, yield to the pernicious contagion of example, or to the hard pressure of competition forced upon them by their less scrupulous neighbours.

"The adulteration of drinks deserves special notice, because your committee cannot but conclude that the intoxication so deplorably prevalent is in many cases less due to the natural properties of the drinks themselves than to the admixture of narcotics, or other noxious substances, intended to supply the properties lost by dilution.

"The Committee desire to leave the execution of the law against adulteration in the hands of the local authorities, but they are of opinion that very valuable assistance would be afforded to such bodies in ascertaining the fact of adulteration if one or more scientific analysers were to be appointed under the authority of the General Board of Health, to whom the local authorities might, whenever they thought fit, refer any articles seized under suspicion of adulteration for analysis, and who would thus enable the persons charged with the administration of the law to obtain at once, and without cost, a fully competent opinion in all difficult cases.

"These analysers should also undertake to examine any articles sent to them by private individuals, on payment of the expenses of such examination.

"With regard to patent medicines, there can be no doubt that the public health is endangered by the use of several of these compounds; and your Committee are of opinion that the stamp duty, by giving them a seeming Government sanction, has an injurious influence in encouraging their sale and consumption, and should be abandoned whenever this can be done with a due regard to the wants of the public revenue."

